# Spectral characterization and severity assessment of rice blast disease using univariate and multivariate models

**DOI:** 10.3389/fpls.2023.1067189

**Published:** 2023-02-23

**Authors:** Nandita Mandal, Sujan Adak, Deb K. Das, Rabi N. Sahoo, Joydeep Mukherjee, Andy Kumar, Viswanathan Chinnusamy, Bappa Das, Arkadeb Mukhopadhyay, Hosahatti Rajashekara, Shalini Gakhar

**Affiliations:** ^1^ Division of Agricultural Physics, Indian Agricultural Research Institute, Indian Council of Agricultural Research (ICAR), New Delhi, India; ^2^ Division of Plant Pathology, Indian Agricultural Research Institute, Indian Council of Agricultural Research (ICAR), New Delhi, India; ^3^ Division of Plant Physiology, Indian Agricultural Research Institute, Indian Council of Agricultural Research (ICAR), New Delhi, India; ^4^ Natural Resources Management, Indian Agricultural Research Institute, Indian Council of Agricultural Research (ICAR), Goa, India; ^5^ Division of Agricultural Chemicals, Indian Agricultural Research Institute, Indian Council of Agricultural Research (ICAR), New Delhi, India; ^6^ Department of Plant Pathology, Directorate of Cashew Research, Indian Council of Agricultural Research (ICAR), Karnataka, India

**Keywords:** rice blast, hyperspectral remote sensing, JM distance, vegetation indices, ratio blast index, normalized difference blast index, machine learning techniques, support vector machine regression (SVM)

## Abstract

Rice is the staple food of more than half of the population of the world and India as well. One of the major constraints in rice production is frequent occurrence of pests and diseases and one of them is rice blast which often causes yield loss varying from 10 to 30%. Conventional approaches for disease assessment are time-consuming, expensive, and not real-time; alternately, sensor-based approach is rapid, non-invasive and can be scaled up in large areas with minimum time and effort.  In the present study, hyperspectral remote sensing for the characterization and severity assessment of rice blast disease was exploited. Field experiments were conducted with 20 genotypes of rice having sensitive and resistant cultivars grown under upland and lowland conditions at Almora, Uttarakhand, India. The severity of the rice blast was graded from 0 to 9 in accordance to International Rice Research Institute (IRRI).  Spectral observations in field were taken using a hand-held portable spectroradiometer in range of 350-2500 nm followed by spectral discrimination of different disease severity levels using Jeffires–Matusita (J-M) distance. Then, evaluation of 26 existing spectral indices (r≥0.8) was done corresponding to blast severity levels and linear regression prediction models were also developed. Further, the proposed ratio blast index (RBI) and normalized difference blast index (NDBI) were developed using all possible combinations of their correlations with severity level followed by their quantification to identify the best indices. Thereafter, multivariate models like support vector machine regression (SVM), partial least squares (PLS), random forest (RF), and multivariate adaptive regression spline (MARS) were also used to estimate blast severity. Jeffires–Matusita distance was separating almost all severity levels having values >1.92 except levels 4 and 5. The 26 prediction models were effective at predicting blast severity with R^2^ values from 0.48 to 0.85. The best developed spectral indices for rice blast were RBI (R1148, R1301) and NDBI (R1148, R1301) with R^2^ of 0.85 and 0.86, respectively. Among multivariate models, SVM was the best model with calibration R^2^=0.99; validation R^2^=0.94, RMSE=0.7, and RPD=4.10. The methodology developed paves way for early detection and large-scale monitoring and mapping using satellite remote sensors at farmers’ fields for developing better disease management options.

## Introduction

As the population is booming, the demand for food will surge. It is essential to raise crop efficiency to increase the food supply in order to meet this enormous demand for food. Cereals are a reliable source of staple food for people. Among them, rice is one of the most common and widely cultivated all over the world, particularly in Asia and Africa. However, diseases are the major biotic stresses to rice that results in significant quality and yield losses (Skolik et al., 2018; Yang et al., 2019). Among all the diseases that have an impact on rice growth, rice blast has long been recognized as one of the most devastating diseases because of its worldwide distribution and the resulting severe yield loss (Zhang et al., 2003). The extent of damage depends on several environmental factors, resulting in losses of 10–30% of the global yield of rice. Therefore, disease prevention and treatment are essential components for rice growth and development.

Rice blast is caused by *Pyricularia grisea* (synonymous *Pyricularia oryzae* Cav.; teleomorph *Magnaporthe grisea*). *Pyricularia oryzae* is the most studied species in the Magnaporthales and was ranked first among the top 10 fungal plant pathogens in the world based on the scientific and economic significance of the disease it causes on rice (Dean et al., 2012). It affects all plant developmental stages and causes symptoms on the leaf, collar, neck, panicle, and even in the glumes. Leaf blast symptoms often consist of long, diamond-shaped lesions with a brown or reddish-brown edge and a grey or whitish center (Acharya et al., 2019). Cool and rainy conditions, water stress in the nursery, and excessive nitrogen field dressing all contribute to the disease’s growth. The fungus thrives on rice straws, weeds, reeds, rushes, and cereals that resemble millet. When rice seedlings are seeded, the fungus spreads its spores and infects them (Shahriar et al., 2020).

Traditionally, specialists or seasoned farmers rely on visual and manual skills to identify rice diseases. The development of molecular biology and the associated techniques has also allowed for the accurate detection of rice diseases, and these methods such as (enzyme-linked immune sorbent assay (ELISA), polymerase chain reaction (PCR), etc. are commonly accepted as “standard” or “reference” methods in the domains they are related to (Feng et al., 2020). However, these strategies also possess several drawbacks such as being time-consuming, cost-ineffectiveness, etc. that limit their large-scale acceptance as well as applications. Therefore, a non-destructive detection system of blast disease could be effective to support the judicious application of crop protection chemicals at an appropriate place, and time at appropriate dosages and thereby can assist farmers to control the cost of production which also pertains to sustainable agri-input management. Recent advancements in optical sensor technology may make it possible to detect foliar diseases directly in the field (West et al., 2003). Additionally, because of its great spectral resolution, hyperspectral technology has been increasingly popular in recent years for crop pest and disease monitoring. It may detect unseen signs representing the physiological status of plants at the early stages of disease (Bongiovanni and Lowenberg-Deboer, 2004; Haboudane, 2004; Inoue et al., 2012). In a study by Lin et al. (2020), different reflectance-based vegetation indices were identified for the assessment of rice sheath blight. Similarly, Yudarwati et al. (2020) found the vegetation indices consist of blue, green, red, and red edge bands normalized green red difference index (NGRDI), normalized pigment chlorophyll ratio index (NPCI) and plant senescence reflectance index (PSRI) exhibit higher predicting power for BLB infestation. Liu et al. (2008) gathered rice reflectance spectra in the laboratory and field conditions and used the reflectance ratio to determine the severity of the brown rice spot disease. Moreover, for the first time, Yang (2012) investigated the near-infrared hyperspectral image to detect blast rice in N, Kipponbare at the seedling stage.

Furthermore, the only way to obtain more comprehensive information on crop disease is to look into the entire spectrum of electromagnetic radiation in the optical range. Using the hyperspectral data in a completer optical range has a problem with high dimensionality and significant multi-collinearity in the linked data (Vaiphasa et al., 2007; Sahoo et al., 2015). Hyperspectral data-based multiple linear regression (MLR) models typically suffer from multi-collinearity and are over-fitted since the number of observations may be equal to or less than the predictors (Curran, 1989). Partial least square regression (PLSR) and MLR can effectively restrain the problems of multi-collinearity and overfitting (Cho et al., 2007). Ban et al. (2019) estimated the apple scab disease severity and correlated it with the SPSD chlorophyll reading with the help of multivariate modeling, PLSR. Ahmed et al. (2019) have also studied different machine learning algorithms including that of KNN (K-Nearest Neighbour), J48 (decision tree), naive bayes, and logistic regression, decision tree algorithm for crop disease. Liang et al. (2019) investigated a deep convolutional neural network for blast disease recognition. There are very few studies to investigate vegetation indices and machine learning techniques for blast disease assessment and to the best of our knowledge, there is no study where a comparison of various multivariate models has been done for the assessment of disease severity using a diverse set of rice genotypes.

In the present study, a total of 26 vegetation indices were evaluated for the rice blast disease. Additionally, two indices (RBI, NDBI) were also proposed for the assessment of rice blast severity. The ratio blast index (RBI) and normalized difference blast index (NDBI) were obtained after analyzing all possible combinations between 350-2500nm and their correlations with blast severity (Das et al., 2017). Moreover, rice blast severity was estimated from the reflectance spectra using PLS along with multivariate adaptive regression spline (MARS), support vector machine regression (SVM), and random forest (RF). When the relationship between the spectra and the qualities to be modeled is not linear, SVM, MARS, and RF are more appropriate (Ding and Peng, 2012; Bao et al., 2014). Therefore, to access the severity of leaf blasts in rice, a non-destructive spectroscopy-based approach is being developed. Briefly, the objectives of the present study were (i) to compare the effectiveness of reported spectral indices and two proposed indices for high throughput phenotyping of rice blast severity in comparison to the conventional approaches, and (ii) to evaluate the retrieval accuracy of severity assessment using four different multivariate models.

## Materials and methods

### Experimental site

**Figure 1:**
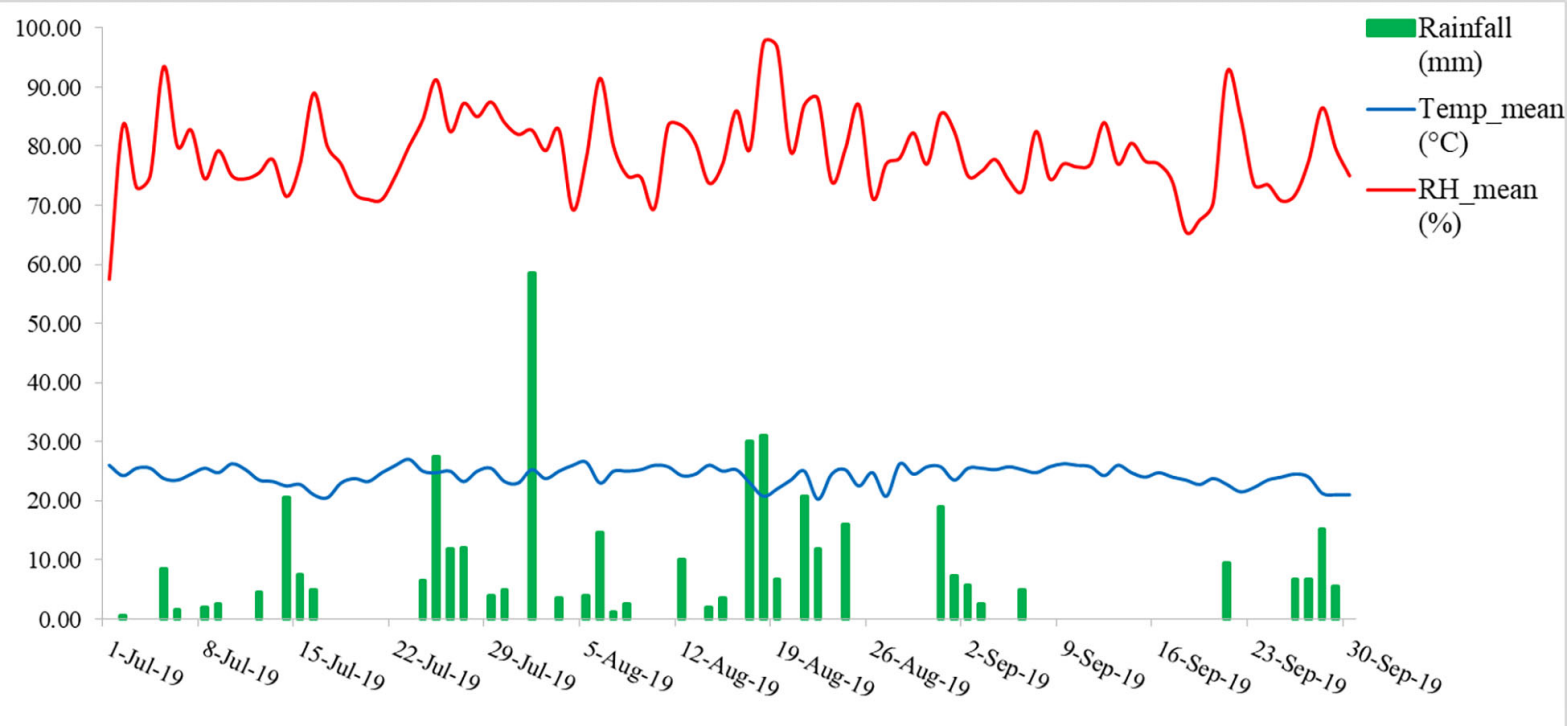
Daily weather condition during the rice growing season.

A field experiment was conducted in the year 2019, at Almora, Uttarakhand, India (29.59°N latitude, 79.64°E longitude, and at an altitude of 1245 m AMSL). The rice was grown under two conditions, one in upland condition and another in lowland condition, with 10 genotypes of rice each both blast sensitive and blast resistant taking 3 replications each laid in a randomized block design (**Table 1**). The meteorological data of the experimental site indicated that daily mean temperature and mean relative humidity during the growing season fluctuated from 20.25 to 26.0°C and 57.5 to 97.5%, respectively. The total rainfall received was 418.5 mm during the growing season (**Figure 1**).

**Table 1:** Genotypes of rice grown under upland and lowland conditions.

Upland condition	Lowland condition
Rice genotype	Rice genotype
VL 32475	DH-94
DSN-119	DH-33
VL 32473	DH-47
DSN-140	Bala
BL-21	DH-34
BL-6	DH-32
DSN-120	DH-30
BL-12	DH-49
BL-10	DH-44
BL-18	DH-79

### Cultivation condition and pathogen inoculation

An isolate of rice blast *M. oryzae* Isolate 1637 was used in the present study. The fungus was maintained through the study by sub-culturing on Rice Straw Extract Agar (RSEA) medium for sporulation and on Potato Dextrose Agar. Spore Suspension of *M. oryzae* isolates 1637 with concentration 106 mL^-1^ prepared and added with Tween 20. The suspension was sprayed with the help of a hand sprayer over the seedlings. As the rice was grown with a total of 20 genotypes of rice posing both blast sensitive and blast resistant, so different level of blast severity has been developed under the favorable condition of Almora, India.

**Figure 2:**
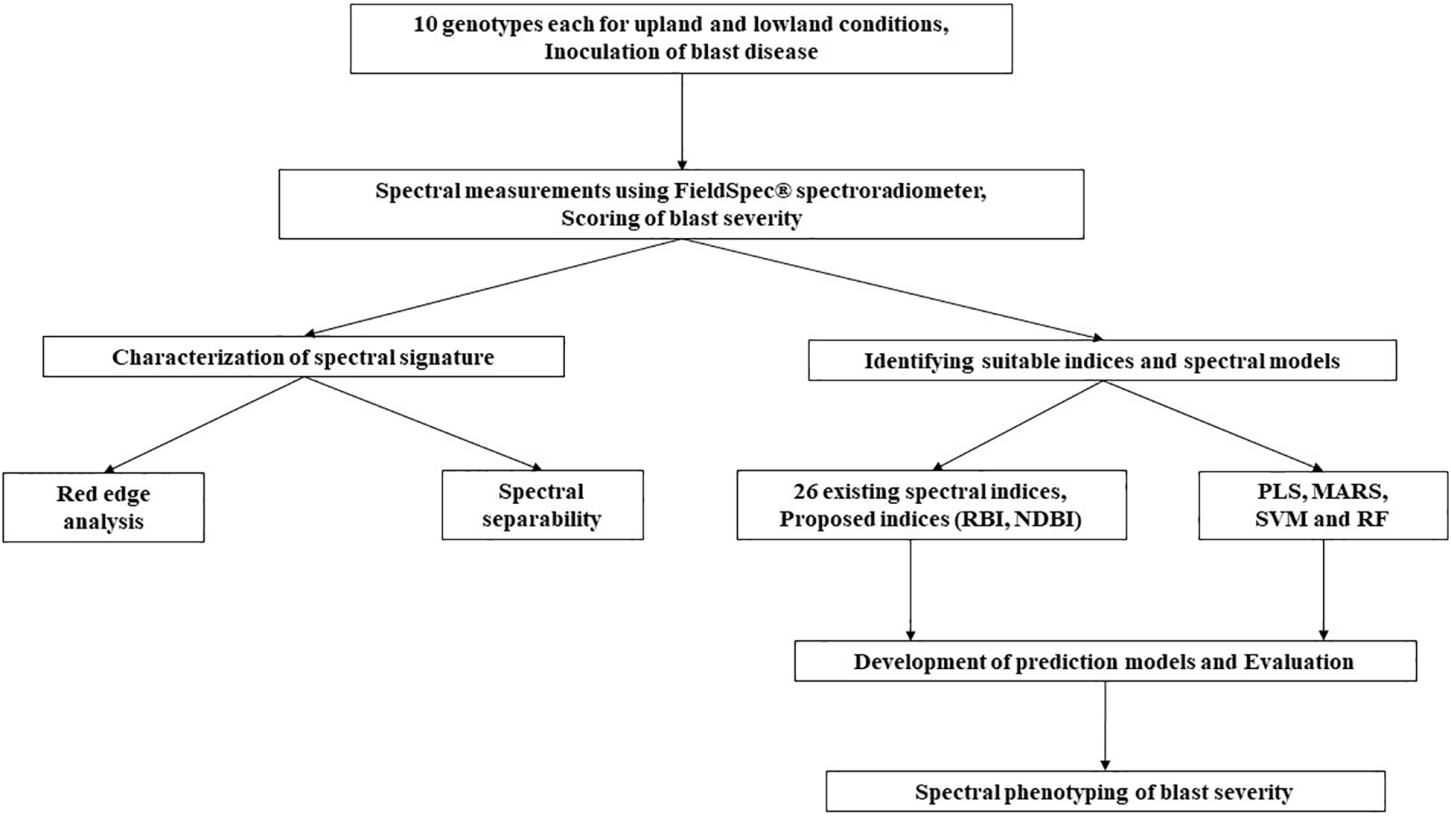
The overall workflow of the present study.

The overall workflow of the present study has been depicted in **Figure 2** and the detailed methodology for each experiment has been depicted in the following subheadings.

### Scoring of blast severity

Its most prevalent and diagnostic symptom is diamond-shaped lesions, which occurs on the leaves, while lesions on the sheaths are very uncommon. Depending on the environment, the age of the plant, and the level of resistance of the host cultivars, the symptoms on leaves may change. On the leaves, tiny brown spots first appear, then they develop into rhombic, elongated, and, occasionally, several cm long patches. The spots have centers that are grayish-white, but when they dry up, they turn straw-colored and form a brownish border (Asibi et al., 2019). Older lesions frequently take on a pale tan color with necrotic edges on cultivars that are vulnerable. In the field, the disease severity level was estimated by evaluating the percentage of the relevant host tissue or organ covered by symptoms (or lesions) of the disease and the number and size of lesions. In this study, the extent of disease severity was evaluated based on the protocols given by the IRRI (International Rice Research Institute) (1996) (**Table 2**).

**Table 2:** Description of different scores of rice blast disease.

Rating	Description
0	No lesion observed
1	Small brown specks of pin point size
2	Small roundish to slightly elongated, necrotic gray spots, about 1-2 mm in diameter, with a distinct Moderately Resistant brown margin. Lesions are mostly found on the lower leaves
3	Lesion type same as in 2, but significant number of lesions on the upper leaves
4	Typical susceptible blast lesions, 3 mm or longer infecting less than 4% of leaf area
5	Typical susceptible blast lesions of 3mm or longer infecting 4-10% of the leaf area
6	Typical susceptible blast lesions of 3 mm or longer infecting 11-25% of the leaf area
7	Typical susceptible blast lesions of 3 mm or longer infecting 26-50% of the leaf area
8	Typical susceptible blast lesions of 3 mm or longer infecting 51-75% of the leaf area many leaves are dead
9	Typical susceptible blast lesions of 3 mm or longer infecting more than 75% leaf area affected

### Spectral reflectance measurement

Canopy reflectance of the field was measured for 10 (0-9 scale) disease severity levels along with 10 different genotypes with help of a hand-held ASD Field Spec spectroradiometer. It operates between 350 and 2500 nm, with sampling intervals of 1.4 nm between 350 and 1050 nm and 2 nm between 1000 and 2500 nm. After resampling, the instrument supplied data with a 1 nm interval. All analyses have relied on spectrum reflectance data with a bandwidth of 1 nm (Need reduced sampling for spectral index development). The spectral measurement was made between the hours of 11:00 a.m. and 1:00 p.m. on a sunny, clear day using the spectroradiometer with a 25° field of view and placed 0.5 meters height from the canopy and at the nadir position. The device was optimized and reference reflectance was taken with a white reference panel called spectralon before canopy reflectance measurements. The average of the sample’s 30 spectral scans is used to calculate each spectral measurement. We have collected 30 spectral signatures for all severity levels from both upland and lowland conditions.

### Data pre-processing

Prior to the data analysis, spectral data are frequently pre-processed to increase the predictive power of multivariate calibration models. This is because variation in the predictor variables unrelated to the response variable may lower the predictive power of the models. Pre-processing is intended to lessen the impacts of random noise and enhance the signal-to-noise ratio. The Savitzky-Golay filter, which employs a moving polynomial fit of any order and whose size is determined as (2n+1) points, where n is the half-width of the smoothing window, is the most widely used filter in spectral data processing. The polynomial fit interpolates the locations between the 2ns (Savitzky and Golay, 1964).

### Spectral derivative and red edge analysis

The mean reflectance’s first derivative was determined. Then, under different levels of disease severity, red edge shifts and shapes in the first derivative curve were examined. By fitting a second-order polynomial equation to the red and infrared slope, the linear interpolation technique was used to determine the amplitude (drre) of the red peak for each infection level (Guyot et al., 1988). The red edge parameters; drre, the amplitude of the red edge peak in the first derivative reflectance curve, and ∑(dr 670-780), the area under the red edge curve were used to characterize the spectrum under various severity levels (Gazala et al., 2013).

### Data processing for spectral discrimination of blast severity

It is hard to utilize all the wave bands from 350-2500 nm for severity level discrimination (n=1763 excluding ambient absorption bands). Due to noise from ambient water absorption, the wavelengths between 1351 and 1439 nm, 1801 and 1949 nm, and 2351 and 2500 nm were excluded from the analysis. Then, 10 nm averaged data was calculated to reduce the data redundancy. The number of relevant wavelengths obtained from the resampling analysis was further reduced using the “Genetic algorithm mixed with PLS regression” (GA-PLS).

### Spectral separability for discriminating blast severity levels

For spectral discrimination of 10 disease severity levels, the best bands from GA-PLS study were employed. Jeffries-Matusita (J-M) distance was taken into consideration as the measure of the spectral separability index (Vaiphasa et al., 2007; Ismail et al., 2008). The average distance between two class density functions is known as the Jeffries-Matusita distance between two probability functions (Richards, 1993; Schmidt and Skidmore, 2003). The square of the J-M distance runs from 0 to 2, and a higher value denotes that the class pairs are more separable (Richards, 2013). Instead of using the threshold value of 1.90 that is typically used in remote sensing datasets, a higher J-M distance of values greater than 1.94 (97% of 2) was evaluated in the current investigation as the threshold of spectral separability between disease severity levels (Das et al., 2020). The J-M distance formula is provided below:


(1) $$\text{J}-{\text{M}}_{\text{mn}}=\sqrt{2\left(1-{\text{e}}^{-\text{α}}\right)}$$

(2) $$\text{α}=\frac{1}{8}{\left({\text{μ}}_{\text{m}}-{\text{μ}}_{\text{n}}\right)}^{\text{T}}{\left(\frac{{\text{C}}_{\text{m}}+{\text{C}}_{\text{n}}}{2}\right)}^{-1}\left({\text{μ}}_{\text{m}}-{\text{μ}}_{\text{n}}\right)+2\text{ln}\left(\frac{\left(\frac{1}{2}\right)\left|{\text{C}}_{\text{m}}+{\text{C}}_{\text{n}}\right|}{\sqrt{\left|{\text{C}}_{\text{m}}\right|\times }\left|{\text{C}}_{\text{n}}\right|}\right)$$

where, J–M_mn_ represents the J–M distance between the severity score pair m and n; μ_m_ is the spectral reflectance’s mean vector of m^th^ severity score at optimum wavebands acquired from GA-PLS analysis; C_i_ = the spectral reflectance’s covariance matrix of m^th^ severity score at optimum wavebands; |C_m_| = the determinant matrix of C_m_; ln = natural logarithm function; T = transpose function of matrices.

### Prediction of blast severity using index-based regression models

In the present study, 26 narrow-band spectral indices were calculated using canopy reflectance at different wavelengths. The equations and the references for these indices have been presented in **Table 3**. Then these indices were correlated with the score of the rice blast. The indices which showed a higher correlation coefficient (r ≥ 0.8) were used to develop linear regression models for disease severity prediction using 2/3^rd^ of the total 600 data. Here, we have compiled all the data from both management conditions to make the model more robust. Then these prediction regression models were validated using spectral indices data for the remaining 1/3^rd^ dataset.

**Table 3:** Details of spectral indices used for regression model development for disease severity prediction.

S. No.	Index	Formula	References
Structural indices			
1	Green Index (GI)	R_554_/R_677_	Zarco-Tejada et al., 2005
2	Green Vegetation Index (GVI)	(R_682_−R_553_)/(R_682_+R_553_)	Kauth and Thomas, 1976
3	Lichtenthaler Indices (Lic1)	(R_790_ – R_680_)/(R_790_ + R_680_)	Lichtenthaler et al., 1996
4	Modified Simple Ratio (MSR)	(R_800_/R_670_ − 1)/[(R_800_/R_670_)0.5 + 1]	Chen, 1996
5	Modified Triangular Vegetation Index (MTVI)	1.2*[1.2*(R_800_-R_500_)2.5*(R_670_-R_550_)]	Haboudane et al., 2002
6	Normalized Difference Vegetation Index (NDVI)	(R_830_ − R_660_)/(R_830_ + R_660_)	Rouse et al., 1973
7	Normalized Difference Water Index (NDWI)	(R_560_ − R_830_)/(R_560_ + R_830_)	McFeeters, 1996
8	Perpendicular Vegetation Index (PVI)	(R_NIR_-αR_red_-b)/(1+α^2^) NIR,soil = α R_red_,soil+b	Richardson and Wiegand, 1977
9	Red Green Index (RGI)	R_690_/R_550_	Zarco-Tejada et al., 2005
10	Renormalized Difference Vegetation Index (RDVI)	R_800_ − R_670_)/[(R_800_ + R_670_)^0.5^]	Roujean and Breon, 1995
11	Second Modified Triangular Vegetation Index (MTVI2)	1.5(1.2*(R_NIR_ – R_green_) – 2.5(R_Red_ – R_green_)]/[(2 R_NIR_+1)2 – (6 R_NIR_ – 5 R_Red_0.5) – 0.5]0.5	Haboudane, 2004
12	Triangular Vegetation Index (TVI)	0.5 * [120 * (R_750_ − R_550_) – 200 * (R_670_ − R_550_)]	Broge and Leblanc, 2001
13	Water Band Index (WBI)	R_900_/R_970_	Penuelas et al., 1995
Biochemical indices			
14	Pigment-Specific Normalized Difference-a (PSNDa)	R_800_/R_680_	Blackburn, 1998
15	Pigment-Specific Simple Ratio-b (PSSRb)	R_800_/R_635_	Blackburn, 1998
16	Pigment-Specific Simple Ratio-c (PSSRc)	R_800_/R_470_	Blackburn, 1998
17	Modified Chlorophyll Absorption Ratio Index (MCARI)	[(R_700_ − R_670_) − 0.2 (R_700_ − R_550_)] (R_700_/R_670_)	Daughtry, 2000
18	Water Band Index (WBI)	R_970_/R_900_	Wang et al., 2007
19	Green Vegetation Index (GVI)	(−0.283MSS4 − 0.66MSS5 + 0.577MSS6 + 0.388MSS7)	Kauth and Thomas, 1976
20	Modified Chlorophyll Absorption Ratio Index (MCARI)	[(R_700_ − R_670_) − 0.2 (R_700_ − R_550_)] (R_700_/R_670_)	Daughtry, 2000
21	Normalized Difference Chlorophyll Index (NDCI)	(R_762_-R_527)_/(R_762_+R _527_)	Marshak et al., 2000
Physiological indices			
22	Moisture Stress Index (MSI)	R_1599_/R_819_	Hunt and Rock, 1989
23	Normalized Difference Infrared Index (NDII)	(R_780_ − R_710_)/(R_780_ − R_680_)	Datt, 1999
24	Photochemical Reflectance Index (PRI)	(R_531_ − R_570_)/(R_531_ + R_570_)	Gamon et al., 1992
25	Photochemical Reflectance Index-2 (PRI2)	(R_531_ − R_570_)/(R_531_ + R_570_)	Gamon et al., 1992
26	Red-edge Vegetation Stress Index (RVSI)	rR_714_+R_752_/2-R_733_	Merton and Huntington, 1999

### Proposed indices

The proposed Ratio Blast Index (RBI) and Normalized Difference Blast Index (NDBI) have the following forms:


(3) $$\text{RBI}=\frac{R\lambda 1}{R\lambda 2}$$

(4) $$\text{NDBI}=\frac{\left(R\lambda 1-R\lambda 2\right)}{\left(R\lambda 1+R\lambda 2\right)}$$

These two indices were computed for every two-pair combination wavelength that was conceivable. Correlation analysis was used to determine the correlation coefficient (r) between the derived indices and the severity level of the blast disease. A matrix plot that displayed a characteristic pattern with a number of hotspots with a reasonably high coefficient of determination was created by plotting all of the squares of r values, which represent the coefficient of determination. The wavelength combinations with the highest coefficient of determination were chosen as the best indices (Lu et al., 2018).

### Multivariate machine learning techniques

#### Partial least square

One of the most prevalent issues in machine learning is multicollinearity. This happens when a dataset’s two or more predictor variables have a high degree of correlation. Partial least square (PLS), a powerful multivariate statistical technique, solves the challenges associated with multicollinearity by concurrently performing principle component extraction and classification (Shen et al., 2011; Ursani et al., 2012; Helmholz et al., 2014). PLS uses component projection to have a reduced number of uncorrelated components (also known as latent variables, or LVs) from the entire spectrum with minimal information loss. The global minimum of the root mean square error of cross validation (RMSECV) was used to calculate the number of LVs (Darvishzadeh et al., 2008). In the current study, “plsr” function of the “pls” package (Mevik and Wehrens, 2007) was performed in R version 3.5.0 to implement PLS.

#### Multivariate adaptive regression splines

A set of splines with various gradients are used in the multivariate adaptive regression splines (MARS) method to statistically fit the data of dependent and independent variables (Friedman, 1991). One set of data ends and another one begins at the extremities of the splines (knots), resulting in piecewise curves known as “basis functions” or “hinge functions”. We utilized the function “earth” in package “earth” to implement this method (Milborrow, 2014) in R version 3.5.0.

#### Support vector machine

It is a margin-based classifier. It is widely used for non-separable data like Vis-NIR spectral data. To maximize the distance to the closest examples that can be neatly divided, the support vector machine (SVM) selects a hyperplane that splits the data into two different classes. To solve the SVM problem, the data is transformed from a complex space with non-linear multivariate relationships into a higher dimensional linear space *via* kernel trick, which is then back-transformed to the lower dimensional space for the output of the results. The SVM approach for regression and function approximation is implemented using support vector regression (SVR) (Smola and Schölkopf, 2004). We employed the “svm” function from package “e1071” (Chang, 2001) in R version 3.5.0.

#### Random forest

Random forest (RF) is used to boost the performance of a single decision tree by fitting numerous trees together to form a “forest,” then integrating the trees using votes from the majority of the prediction trees. Trees that have grown very deeply, in particular, have a propensity to learn highly erratic patterns because they overfit their training sets, which results in low bias but high variance. In order to lower the variance, multiple deep decision trees trained on various subsets of the same training data are averaged using random forests (Hastie et al., 2009). The function “randomForest” from package “randomForest” (Breiman, 2001) was used to implement this technique using R version 3.5.0. RF’s total amount of trees was configured with a default value of 500.

#### Deep neural network

Deep neural network (DNN) is an established artificial neural network system. DNN can be used to train on complex data and foresee outcomes. Single hidden layers are present in the structure of simple neural networks, while many hidden layers are present in DNNs in addition to an input and output layer (Costache et al., 2020). In DNN, the feed-forward network is operated and analysed using the back-propagation function. The network is challenging to train due to the rising number of hidden layers and accompanying varying learning speeds (Tien Bui et al., 2020). Due to the existence of various hidden layers, DNN can tackle a variety of complex classification problems. The DNN algorithm is regarded as a more potent and effective one in neural network systems (Schmidhuber, 2015). We employed “h2o” package (Arora et al., 2015) to implement this method in R version 3.5.0.

The 0-9 levels of disease severity at an interval of 1 were used as response variable for the generation of regression models. At the same time, the ten classes of disease severity levels were used as dependent variables for the development of classification models. The calibration of all the machine learning models has been done using 2/3^rd^ of the total 600 data set whereas the remaining 1/3^rd^ of the data was used for validation purposes. The root mean square error (RMSE), coefficient of determination (R^2^), and residual prediction deviation (RPD) were used to evaluate the regression models’ accuracy.


(5) $$\text{RPD}=\frac{\text{Standard deviation }\left(\text{SD}\right)}{\text{Standard error of prediction }\left(\text{SEP}\right)}$$

(6) $$\text{SEP}=\sqrt{\frac{1}{\text{n}-1}\sum_{i=1}^{n}{\left(\text{Pi}-\text{Oi}\right)}^{2}}$$

(7) $$RMSE=\sqrt{\frac{1}{n}\sum_{i=1}^{n}{\left(P_{i}-O_{i}\right)}^{2}}$$

Where P_i_ is the predicted value, O_i_ is the observed value and n is the number of samples.

Chang et al. (2001) classified prediction accuracies into accurate (RPD > 2), moderate (1.4< RPD< 2), and poor (RPD< 1.4).

The assessment of classification accuracy of different techniques used for the classification was made through confusion or error matrix. The overall accuracy or total accuracy (Ta) was obtained by dividing the total number of correct predictions to the total number of tested predictions as suggested by Lillesand et al. (2000), p. 724. Another coefficient that was estimated from the confusion matrix function was the Kappa coefficient (K) which denoted the degree to which the percentage correct estimations of a confusion matrix due to “genuine” agreement versus “chance” agreement was made. It ranged from 0 (worst) to 1 (best). The formulae of these parameters are (Hasmadi et al., 2009):


(8) $$\text{Overall accuracy}/\text{Total accuracy }\left(\text{Ta}\right)=\frac{\text{Number of correct predictions}}{\text{Total number of predictions}}$$

(9) $$\text{Kappa coefficirnt }\left(\text{K}\right)=\frac{{\text{θ}}_{1}-{\text{θ}}_{2}}{1-{\text{θ}}_{2}}$$

(10) $${\text{θ}}_{1}=\;\frac{\sum_{\text{i=j}=1}^{\text{N}}{\text{x}}_{\text{ij}}}{\text{N}}$$

(11) $${\text{θ}}_{2}=\;\frac{\sum_{i=1}^{\text{N}}{\text{x}}_{\text{i+}}{\text{x}}_{+1}}{{\text{N}}^{2}}$$

Where, x_ij_ = number of counts in the ij^th^ cell of the confusion matrix, N = total number of counts in the confusion matrix, x_i+_ = marginal total of row i, and x_+i_ = marginal total of column i.

## Results

### Rice blast scoring

**Figure 3:**
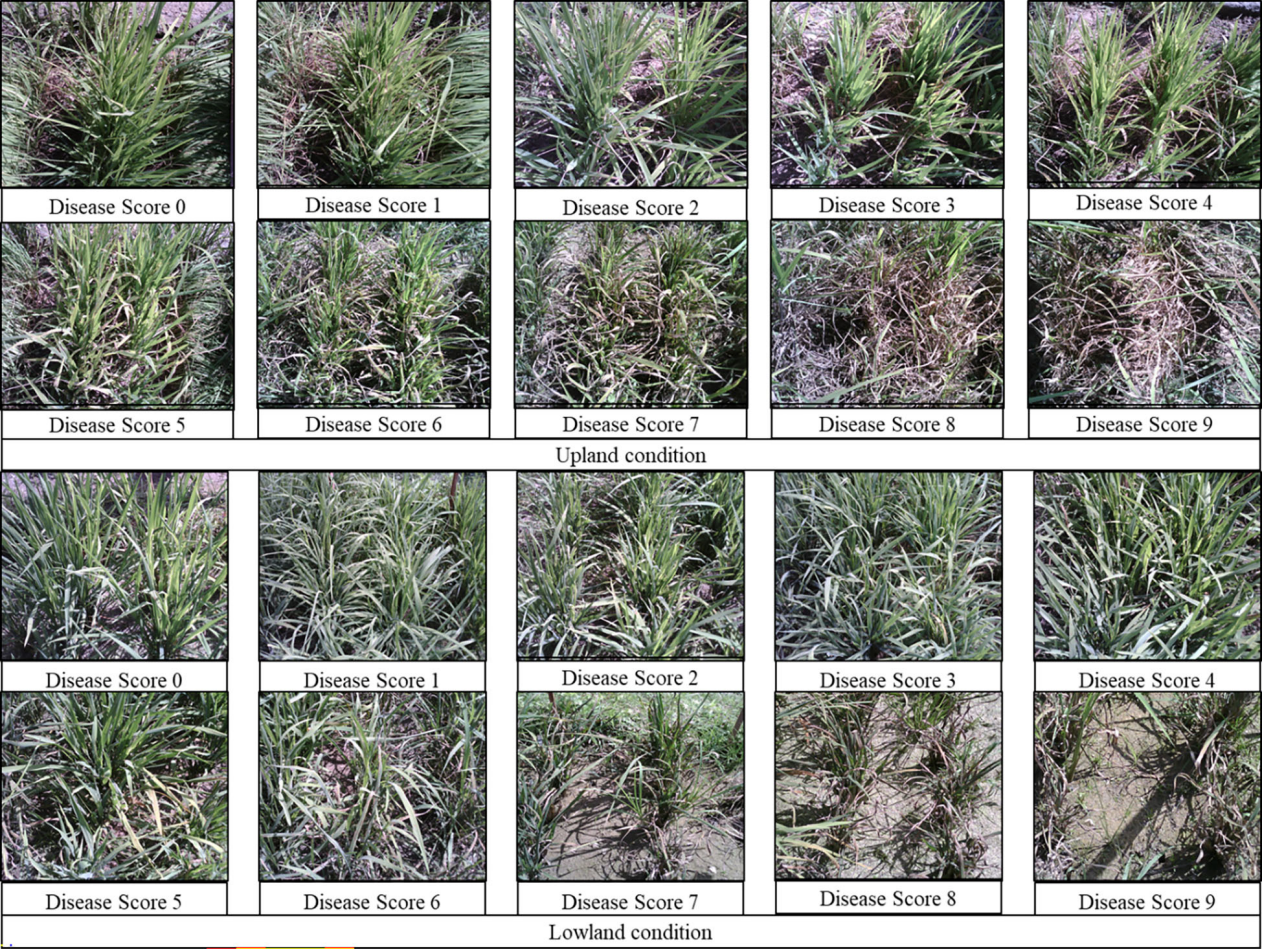
Different severity levels of rice blast infestation under upland and lowland conditions.

The percentage of host tissue covered by the disease’s necrotic lessons, as well as the number and size of the lessons, were evaluated to determine the disease severity levels. According to IRRI guidelines, the severity of the rice blast was rated on a scale of 0 to 9. The plant is shown to be healthy and symptom-free at severity level 0, while the plant is shown to be badly damaged by pathogens at severity level 9. Based on the resistance and susceptibility of the cultivars to the blast disease, the severity levels range from level 0 to level 9, with varying levels of infestation shown in between (**Figure 3**). Rice genotypes, BL 18 and DH 79 grown under upland and lowland conditions, respectively were assigned as level 9. Genotypes, VL 32475 and DH 94 grown under upland and lowland conditions, respectively were assigned as severity level 0. The other cultivars with corresponding severity levels are shown in **Table 4**.

**Table 4:** Name of different genotypes of rice for both upland and lowland conditions.

Upland condition		Lowland condition	
Rice genotype	Disease score	Rice genotype	Disease score
VL 32475	0	DH-94	0
VL 32473	1	DH-47	1
BL-12	2	DH-49	2
BL-10	3	DH-44	3
BL-6	4	DH-32	4
BL-21	5	DH-34	5
DSN-119	6	DH-33	6
DSN-120	7	DH-30	7
DSN-140	8	Bala	8
BL-18	9	DH-79	9

### Effect of disease severity on canopy reflectance and red edge region

**Figure 4:**
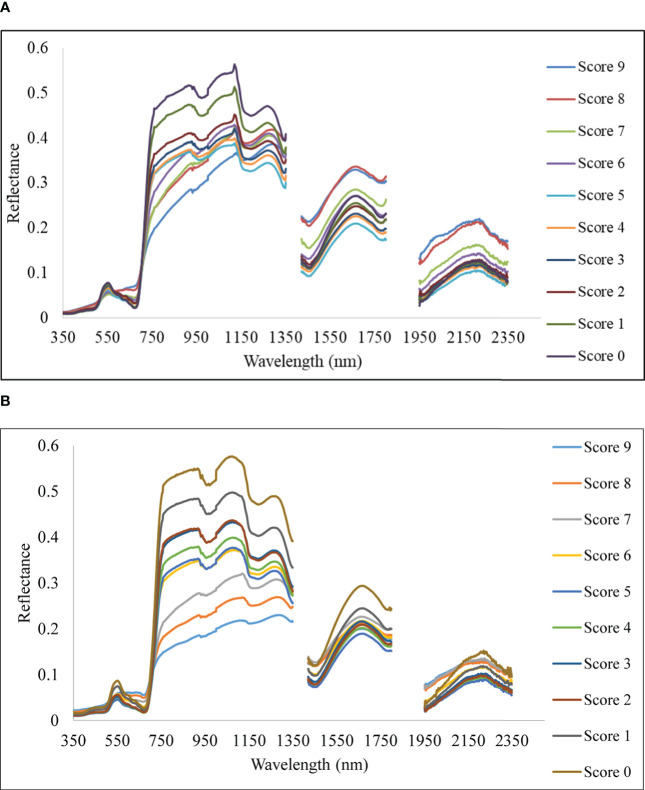
Spectral reflectance of rice canopy under different disease severity levels. **(A)** Upland and **(B)** lowland conditions.

**Figure 5:**
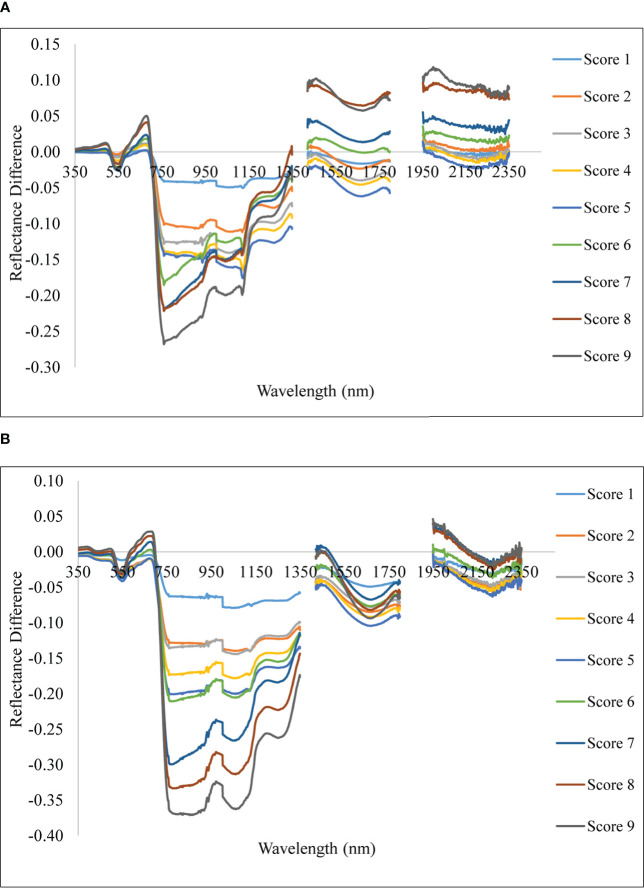
Spectral reflectance difference of rice plant with different severity levels with reference to healthy. **(A)** Upland and **(B)** lowland conditions.

With differing levels of disease infestation, a different spectral response was seen in this study. The dynamic changes in leaf reflectance at various disease infection levels under both conditions are depicted in **Figure 4**. The difference in reflectance between the healthy plant and the rice plant at various severity levels (scores 1 to 9) was calculated and plotted over the spectral range of 350 to 2500 nm (**Figure 5**). The red band, which is around 690 nm, and the NIR, which spans from 800 to 1100 nm, is the spectral ranges where differences are most obvious. The more the severity, the more the positive difference in the red band and the more negative difference in the NIR range. When compared to the disease-infected plant, the seriously afflicted plant had higher reflectance in the short-wave infrared (SWIR) area.

**Figure 6:**
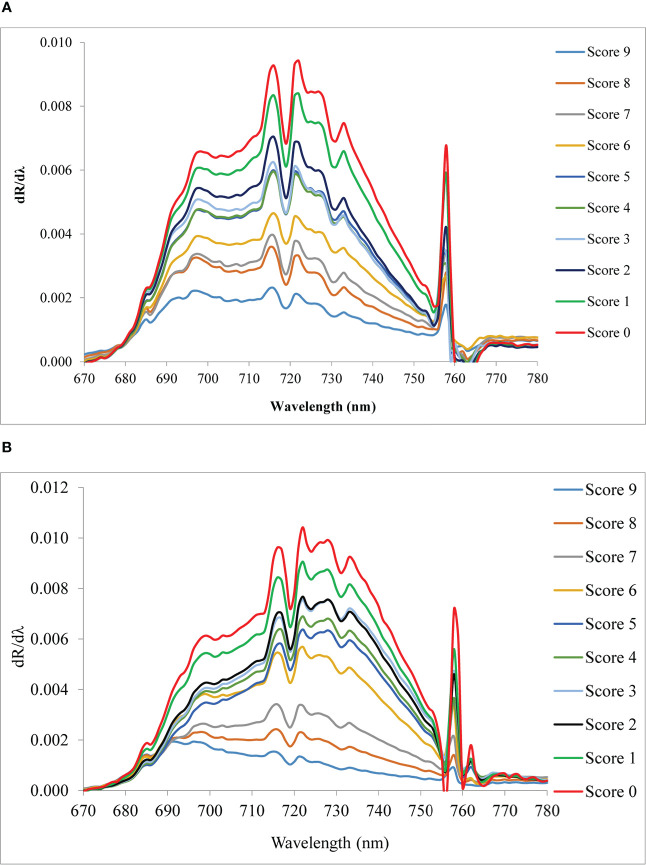
Red edge curve of blast infected rice canopy under **(A)** upland and **(B)** lowland conditions.

**Figure 7:**
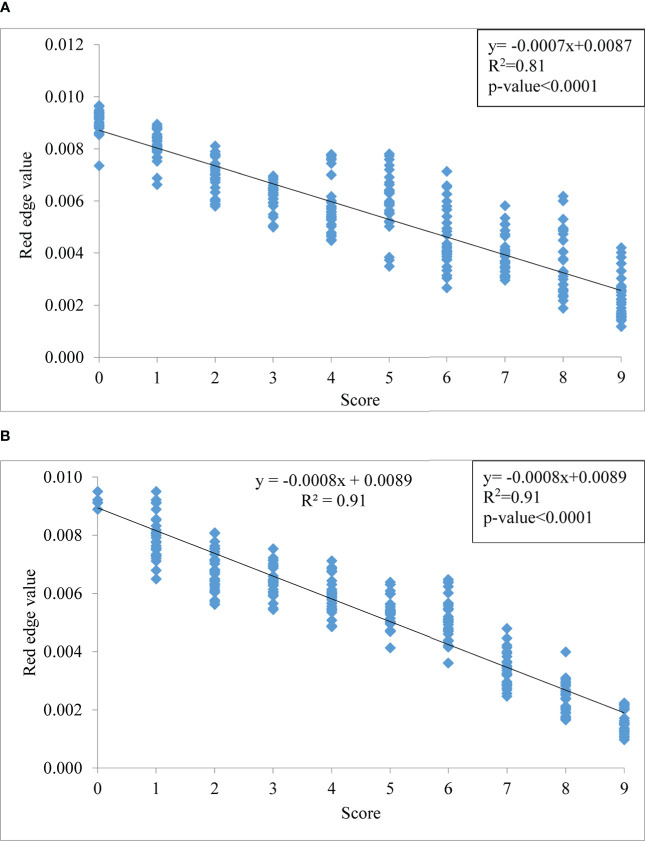
Relationship between disease severity levels and red edge value (REV) under **(A)** upland and **(B)** lowland conditions.

The red edge region is a spectral range from 680 to 760 nm (**Figure 6**). The rate of change of reflectance with the wavelength in the red edge region is very sensitive for the detection of the stress of a crop. The point of inflection where the rate of change of reflectance changes from positive to negative is called the red edge position (REP). There was no evidence of a redshift (i.e shifting of REP to a higher wavelength) with the increase in blast severity levels in this study. However, the amplitude of the red edge peak (red edge value) significantly decreases with the increase in severity levels. The amplitude for scores 0 and 9 was 0.009429 and 0.002301, respectively under upland land conditions whereas the amplitude of the score 0 and 9 was 0.010421 and 0.001935, respectively for lowland rice (**Table 5**). The regression analysis between REV and disease severity score showed a high R^2^ value of 0.81 and 0.91 for upland and lowland conditions, respectively (**Figure 7**). Moreover, it has been observed from **Table 5** that the sum of the first derivative reflectance between 670-780 nm gradually decreases towards the highest disease severity level.

**Table 5:** Effect of blast severity on red edge characteristics.

Disease score	Upland condition		Lowland condition	
	Amplitude of red edge peak (REV)	Area under the red edge curve between 670 and 780 nm	Amplitude of red edge peak (REV)	Area under the red edge curve between 670 and 780 nm
0	0.009429	0.45032	0.010421	0.49294
1	0.008400	0.40711	0.009063	0.43803
2	0.007041	0.33868	0.007677	0.37004
3	0.006242	0.31344	0.007551	0.37090
4	0.005954	0.30184	0.006897	0.33602
5	0.005993	0.30546	0.006378	0.31054
6	0.004648	0.25566	0.005692	0.28623
7	0.003948	0.21308	0.003414	0.18379
8	0.003579	0.19416	0.002420	0.14089
9	0.002301	0.14037	0.001935	0.09902

### Spectral separability using J-M distance analysis

Significant spectral features through the GA-PLS technique were used for spectral separability calculation. To lessen the data redundancy for usage with GA-PLS, spectral averaging was carried out to 10 nm. This method revealed that there were 22 selected wavebands, specifically, 5 in the visible region (380, 400,470, 550, and 600 nm), 8 in the NIR region (730, 740, 900, 1100, 1140, 1180, 1260, and 1290 nm), and 9 in the SWIR zone (1320, 1440, 1480, 1590, 1650, 1780, 1950, and 2020 nm). These wavelengths were used to calculate the J-M distance, which expressed that, except for levels 4 and 5, all severity levels were determined to be separable with J-M distance values of more than 1.94 (97% of 2) (**Table 6**).

**Table 6:** The J-M distance between all 10 disease scores for spectral separability using bands selected from GA-PLS.

Score	0	1	2	3	4	5	6	7	8	9
0	–	1.9809	1.9996	1.9999	1.9999	1.9999	2	2	2	2
1	–	–	1.9709	1.9936	1.9993	1.9968	1.9999	2	2	2
2	–	–	–	1.9494	1.9982	1.9775	1.9998	2	2	2
3	–	–	–	–	1.9806	1.9638	1.9989	2	2	2
4	–	–	–	–	–	1.9285	1.9968	2	1.9999	2
5	–	–	–	–	–	–	1.9964	1.9999	1.9999	2
6	–	–	–	–	–	–	–	1.9866	1.9980	1.9999
7	–	–	–	–	–	–	–	–	1.9978	1.9998
8	–	–	–	–	–	–	–	–	–	1.9851
9	–	–	–	–	–	–	–	–	–	–

### Disease severity and spectral indices

The evaluation of the previously reported spectral indices revealed that the structural indices performed the best for predicting the disease severity levels (**Table 7**). The linear regression models showed the strongest correlations of blast disease with TVI (R^2^= 0.84 and 0.85 for calibration and validation, respectively) with an RPD of 2.52. The PSSRb-based regression model showed the least correlation with the disease severity (R^2^= 0.56 and 0.57 for calibration and validation data sets) with an RPD value of 1.53.

**Table 7:** Regression model for disease prediction using spectral indices.

S. No.	Index	Calibration equation	R^2^ (cal)	R^2^ (val)	RMSE	RPD
Structural indices						
1	GI	Y=-2.6675X+10.251	0.75	0.78	1.36	2.11
2	GVI	Y=11.57X+7.563	0.75	0.78	1.36	1.36
3	Lic1	Y=-15.58X+16.59	0.68	0.68	1.63	1.76
4	MSR	Y=-16.28X+17.38	0.67	0.67	1.64	1.74
5	MTVI	Y=-13.38X+11.01	0.83	0.85	1.19	2.48
6	NDVI	Y=-17.10X+18.17	0.67	0.67	1.66	1.73
7	NDWI	Y=-28.63X+3.928	0.72	0.70	1.57	1.82
8	PVI	Y=-189.7X+2.359	0.83	0.83	1.19	2.41
9	RGI	Y=-7.413X-1.957	0.69	0.70	1.58	1.81
10	RDVI	Y=-19.38X+13.99	0.81	0.83	1.19	2.39
11	MTVI2	Y=15.05X+11.79	0.77	0.79	1.30	2.20
12	TVI	Y=-0.362X+10.78	0.84	0.85	1.17	2.52
13	WBI	Y=-52.84X+58.42	0.72	0.71	1.55	1.85
Biochemical indices						
14	PSNDa	Y=-15.88X+16.87	0.68	0.68	1.63	1.75
15	PSSRb	Y=-0.491X+8.954	0.56	0.57	1.87	1.53
16	PSSRc	Y=-0.338X+10.22	0.63	0.60	1.48	1.55
17	MCARI	Y=-11.58X+10.92	0.82	0.84	1.13	2.53
18	WBI	Y=-52.97X+58.55	0.72	0.71	1.55	1.81
19	GVI	Y=11.57X+7.563	0.75	0.77	1.36	2.39
20	MCARI	Y=-23.79X+8.486	0.71	0.73	1.47	1.94
21	NDCI	Y=-26.24X+23.06	0.51	0.48	2.07	1.38
Physiological indices						
22	MSI	Y=8.660X-1.537	0.69	0.65	1.51	1.89
23	NDII	Y=-14.90X+6.926	0.72	0.70	1.57	1.82
24	PRI	Y=51.84X+4.484	0.67	0.67	1.65	1.73
25	PRI2	Y=50.03X+6.148	0.70	0.71	1.56	1.83
26	RVSI	Y=299X+10.11	0.78	0.79	1.30	2.19

### Proposed ratio blast index and normalized difference blast index

**Figure 8:**
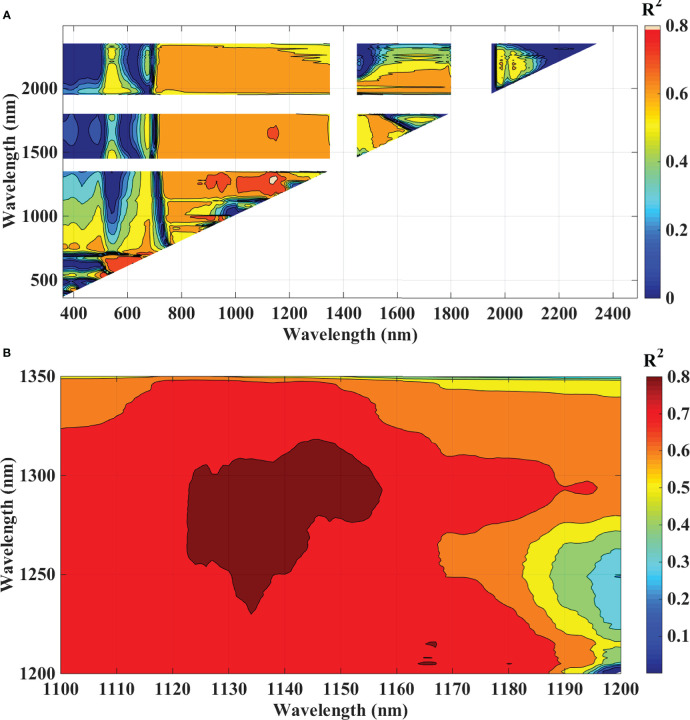
Contour maps of coefficients of determination (R^2^) for linear relationship between blast indices (RBI) with disease scores, of rice canopy under different blast disease severity levels at **(A)** 10 nm sampling interval at 350-2500 nm and **(B)** 1 nm sampling interval.

**Figure 9:**
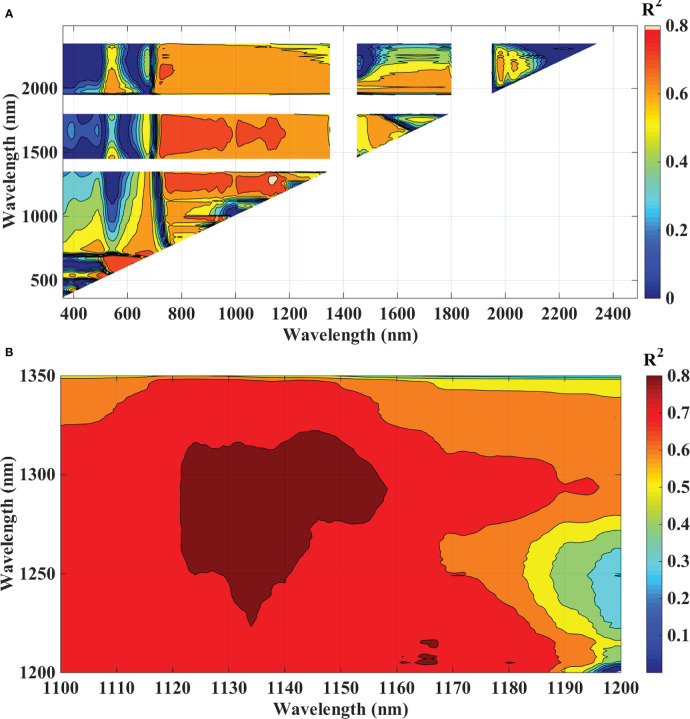
Contour maps of coefficients of determination (R^2^) for linear relationship between blast indices (NDBI) with disease scores, of rice canopy under different blast disease severity levels at **(A)** 10 nm sampling interval at 350-2500 nm and **(B)** 1 nm sampling interval.

To analyze the association between blast severity and the proposed indices in this study namely Ratio Blast Index and Normalized Difference Blast Index from the spectral range of 350 nm to 2500 nm, the reduced sampling approach was employed (Yao et al., 2010) at 10 nm intervals based on leaf spectral reflectance and to identify the sensitive band ranges with higher R^2^ values for the 2/3^rd^ calibration data sets. In the NIR region, several “hotspots” with high blast severity correlation coefficients with RBI and NDBI were found (**Figure 8A** and **9A**). More accurate contour maps of R^2^ values between blast severity with RBI and NDBI at 1 nm intervals were produced by precisely sampling these sensitive spectral regions (**Figure 8B** and **9B**. Based on the R^2^ values, the best bands selected and indices were RBI (R1148, R1301) and NDSI (R1148, R1301) for blast severity. Linear regression equations for blast severity based on RBI and NDBI indices were developed (**Table 8**). The results revealed that the model was the best in predicting blast severity by using both RBI and NDBI values (R^2^= 0.85, RMSE=1.14; R^2^= 0.86, RMSE=1.02, respectively for validation).

**Table 8:** Relationships of disease severity levels to RBI and NDBI.

Spectral index	Regression Equation	R^2^ (cal)	R^2^ (val)	RMSE	RPD
Ratio Blast Index (RBI)	Y=59.211X-54.713	0.85	0.85	1.14	2.52
Normalized Difference Blast Index (NDBI)	Y=177.2X+4.777	0.86	0.86	1.02	2.60

### Multivariate models

**Figure 10:**
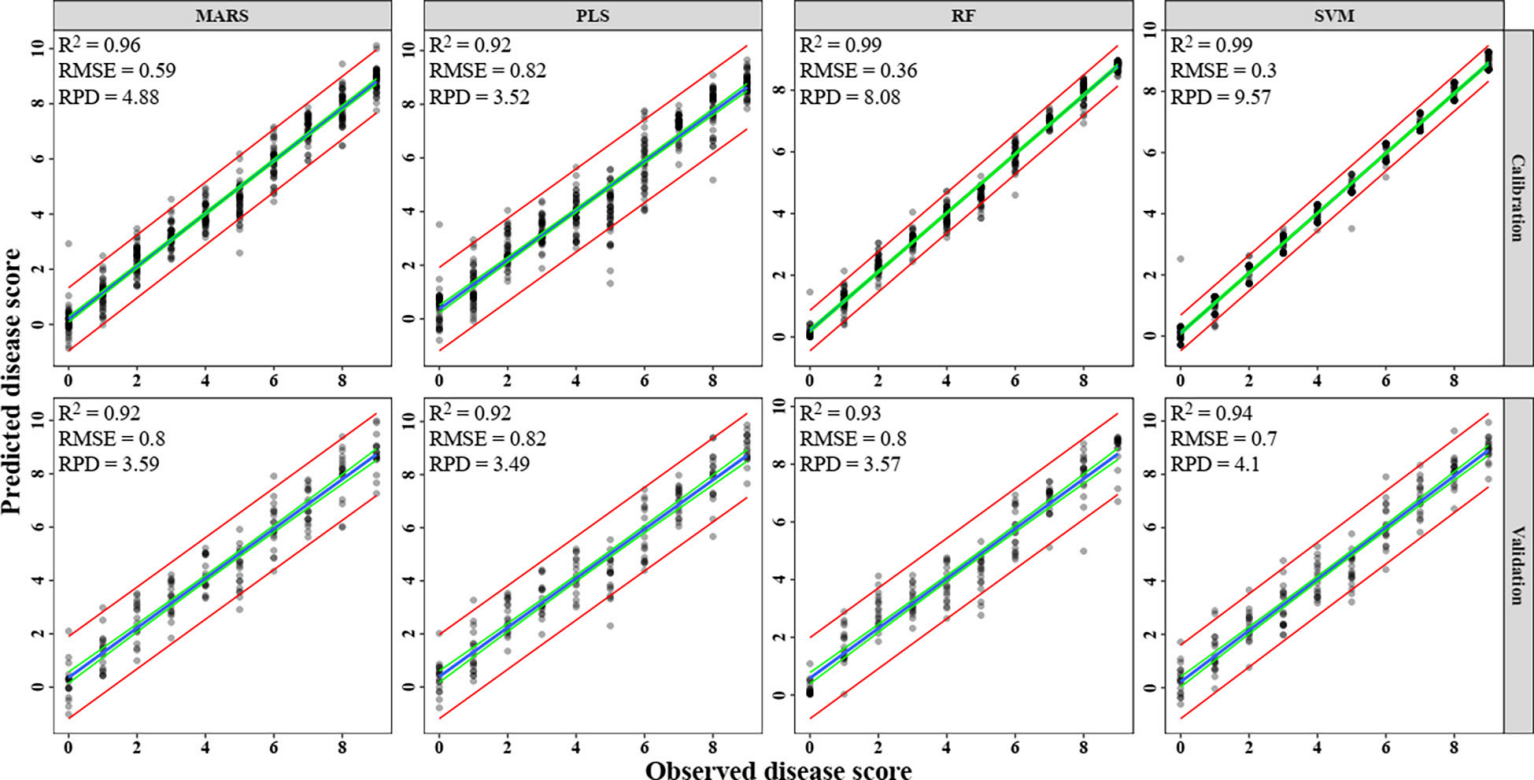
Prediction of rice blast severity levels using multivariate regression models. **(A)** PLS, **(B)** RF, **(C)** MARS, **(D)** SVM.

Spectral reflectance observations were used as independent (X) factors and severity scores as the dependent (Y) variables in the PLS regression model. The RMSE tended to decline as the PLS model’s latent variable count (LV) increased. Over-fitting, however, could result from the inclusion of too many latent variables. The global minimum in cross-validated root mean squared error (RMSECV) suggested that models with up to 20 LVs were the most ideal. For all four multivariate models, the R^2^ value was 0.92, 0.99, 0.96, and 0.99 in the calibration, and in the validation, it was 0.92, 0.93, 0.92, and 0.94 with RMSE of 0.82, 0.80, 0.80 and 0.70, respectively for PLS, RF, MARS, and SVM (**Figure 10**). Among these models, SVM found to be the most accurate and PLS to be the least accurate for prediction of blast severity with respect to RPD of validation (RPD=4.10 and 3.49, respectively). The performance order of the test multivariate models in comparison to RPD is SVM> MARS> RF> PLS (**Figure 10**). The adoption of multivariate approaches increased the accuracy of the blast assessment, according to a comparison of the results of an index-based and multivariate approach. These methods revealed better sensitivity to changes in disease severity since they included the entire spectrum as opposed to the one or two variables employed in the index-based approach.

The classification of rice blast severity levels, using PLS, RF, MARS, SVM and DNN methods were validated using the two most sophisticated statistical techniques. The result of all the model accuracy is presented in **Table 9**. The result shows that, RF model was the most optimal during both calibration and validation with Ta of 0.995 and 0.606 respectively, followed by MARS (calibration Ta = 0.991, validation Ta = 0.567), PLS (calibration Ta = 0.748, validation Ta = 0.544), DNN (calibration Ta = 0.622, validation Ta = 0.535), and SVM (calibration Ta = 0.500, validation Ta = 0.439). The performance order of multivariate models with respect to kappa value was RF>MARS>PLS>DNN>SVM. The performance of PLS, MARS, DNN and RF were found optimum during calibration whereas they performed poorly during validation with less overall accuracy and kappa< 0.6. But when regression was performed, all the models performed well during both calibration and validation (R^2^ > 0.9).

**Table 9:** Classification of rice blast severity levels using different machine learning models.

	Calibration		Validation	
	Accuracy	Kappa	Accuracy	Kappa
**PLS**	0.748	0.720	0.544	0.494
**RF**	0.995	0.995	0.606	0.562
**MARS**	0.991	0.989	0.567	0.519
**SVM**	0.500	0.444	0.439	0.377
**DNN**	0.622	0.579	0.535	0.485

## Discussion

In the present study, the disease severity levels were estimated by evaluating the percentage of host tissue covered by the necrotic lessons of the disease and the number and size of the lesson. The severity level 0 depicts that the plant is healthy having no symptoms at all, whereas the disease severity level 9 depicts that the plant is most severely affected by the pathogen. Due to very high susceptibility towards blast, BL 18 and DH 79 showed the maximum disease severity whereas the resistant variety VL 32475 and DH 94 exhibited the healthy one under upland and lowland conditions respectively. Ordinarily, a plant leaf has a low reflectance in the visible spectral range due to intense chlorophyll absorption and a comparatively high reflectivity in the near-infrared area due to internal leaf scattering and no absorption (Knipling, 1970). With increase in blast severity level, the reflectance in the visible region increased, basically, in the red region, the reflectance is more in the severely affected plant than in the healthy plant. Our result may be attributed to decreased contents of leaf pigment content in response to blast infection which damaged the leaf chlorophyll and caused lower absorption in the visible region. A similar finding was also investigated by Kobayashi et al. (2003) and Zhang et al. (2020). In our study, the reflectance of a healthy plant in the NIR region was higher than that of an infected one and with the increase in disease severity level, the reflectance in the NIR region gradually decreased which may be attributed to the damage of internal leaf structure in response to blast pathogen. This trend is consistent with the findings of Zhang et al. (2020), and Knipling (1970). The fungus generates enormous turgor pressure inside the leaf cell and a thin penetration peg pierces the rice leaf surface using this pressure to enter into the host by damaging the epidermal and mesophyll cell. Due to the severe infection by the pathogen, the plant eventually starts producing reactive oxygen species such as hydrogen peroxide and deposition of callose at the site of infection (Thordal-Christensen et al., 1997; Nishimura et al., 2003) which are the main causes of producing necrotic lesions leading to cell damage and finally death of the plant. Our results also showed higher reflectance in the SWIR region for the severely affected plant as compared to the disease-uninfected plant. This may be attributed to the lower leaf water content of the blast-infected plants. For severely affected plants pathogen almost kills the plant. Therefore, at the disease severity level 9, there is no difference between the spectra of soil and plant as the plant is almost dead. These observations are aligned with results reported by Yang (2012) for blast disease in rice.

Evaluating the red edge region of the spectral reflectance (680-760 nm) from 1^st^ derivative reflectance values, a high correlation of red edge value (REV) with disease scores was found. The pathogen of rice blast damages the cell structure and dries the plant producing necrotic spots. The damage to the chlorophyll content of the plant led to an effect on the red edge value. Cell structure damage and loss of cell water also led to affect the same. The mixed effect of loss of plant chlorophyll and damage to cellular structure yielded this VNIR as most the sensitive region for disease detection. Marín Ortiz et al. (2019) have found the same result for wheat rust disease. The amplitude of the red edge peak decreases with the increase in severity levels. Fahrentrapp et al. (2019) and Adak et al. (2021) reported similar findings for gray mold leaf infections and nitrogen stress in wheat, respectively. The maximum rate of change value at REP is called red edge value (REV) which has good relation with stress levels. The regression analysis between REV and disease severity score showed a high R^2^ value of 0.81 and 0.91 for upland and lowland conditions, respectively. The sum of the first derivative reflectance between 670-780 nm gradually decreased towards the highest disease severity level. This is consistent with findings by Mahlein et al. (2010) who found similar red edge reflection patterns studying sugarbeet leaves infected with fungal diseases such as *C. beticola and U. betae*.

Spectral separability analysis identified 22 bands as spectral features for discrimination. Out of 22 bands, five are in the visible region (380, 400, 470, 550, 600 nm), eight in the NIR region (730, 740, 900, 1100, 1140, 1180, 1260, 1290 nm), and nine in SWIR region (1320, 1440, 1480, 1590, 1650, 1780, 1950, 2020, 2130 nm). This indicates feature bands selected fall broadly into the visible region affected by chlorophyll absorption (Vaiphasa et al., 2007; El-Nahry and Hammad, 2009; Ustin et al., 2009; Das et al., 2018), NIR range affected by cell structure and water content (Schmidt and Skidmore, 2003; Mutanga and Skidmore, 2007; El-Nahry and Hammad, 2009; Ustin et al., 2009; Das et al., 2018) and in SWIR ranges due to variation water and organic compounds, dry matter, lignin, cellulose, and protein, etc. (Majuva-Masafu and Linington, 2006; Teklu et al., 2010). The spectral reflectance increased in the visible region with a decrease in the infrared region and again increased reflectance in the SWIR region with increasing rice blast severity. These may be attributed to decreased contents of pigments in response to *M. oryzae* infection (Kobayashi et al., 2016), which caused the pigments to deteriorate and absorb less efficiently in the visible region. Infection of *M. oryzae* appears to change the internal cellular structure of leaves, thereby altering the spectral reflectance in the NIR region (Knipling, 1970) whereas the lower leaf water content in blast infected leaf tend to increase the reflectance in the SWIR region. Different severity levels were found to be well separable as significant biochemical changes are affecting the whole range of the spectrum. Severity levels of 4 and 5 could not be found separable and the possible reason might be that plant stress effect on biochemical changes at these two severity levels were not having a significant difference or else they did not yield a significant change in spectral value in selective feature bands.

In this research, the evaluation of the existing indices revealed that the structural indices performed the best for predicting disease severity levels. Regarding the structural indices, it mainly takes care of the reflectance in the infrared region which is affected to the maximum extent due to leaf cell damage caused by the pathogen. Out of all the indices TVI was the best-suited indices for rice blast indicated by R^2^ and RPD of 0.85 and 2.52, respectively. TVI is calculated as the areas of a hypothetical triangle in spectral space that connects green peak reflectance minimum chlorophyll absorbance and reflectance at the NIR region. Therefore, these indices are influenced by the chlorophyll content and greenness of the plant, and the internal structure of the leaf. Becoming a necrotic pathogen damages chlorophyll and cell structure, TVI could be good for disease severity measurement considering the nature of the disease. Similarly, Zhang et al. (2012) and Ashourloo et al. (2014) proposed TVI as an effective index for the prediction of wheat rust. The proposed indices in this study, RBI and NDBI were developed using a contour mapping approach. The contour mapping approach has the advantage of providing an efficient selection of the optimal combination of wavebands for development of the effective spectral indices. Based on the highest correlation value, two wavebands namely 1148 nm and 1301 nm were chosen to develop RBI and NDBI and these two indices outperformed the existing indices for the prediction of blast severity. These two wave bands correspond to unique interaction of reflectance at these wavelengths with cell structure variation at different severity levels and water and organic compounds like cellulose effect on spectral reflectance in diseased plants.

Previously, the traditional approach for disease assessment has been based on the vegetation indices (Gitelson et al., 2001; Gitelson and Merzlyak, 1996; Haboudane et al., 2002) and then used some form of a learning algorithm in these feature spaces, especially in the visible region. The development of machine learning techniques in biotic stress assessment has made tremendous progress in the past few years (Ferentinos, 2018; Barbedo, 2019; Lin et al., 2019). However, the development of these approaches depended heavily on the reflectance in the visible region. In the present study, four machine learning techniques (PLS, MARS, RF, and SVM) were evaluated to develop a disease prediction model using the entire spectrum of vegetation (350-2500 nm). All four models had better predictive accuracy with respect to R^2^, RMSE, and RPD values of the validation data set as compared to the index-based models. The SVM was found to be the best with the highest R^2^= 0.94, least RMSE of 0.7, and highest RPD of 4.10 compared to others for the assessment of blast severity. Being a margin-based classifier, SVM is widely used for non-separable data like Vis-NIR spectral data. Prasad et al. (2012) investigated SVM as a predicting technique for different diseases like Tikka (groundnut), rust, powdery and downy mildew (apple), late blight, and early blight (tomato, potato) with an overall accuracy of 89%. Similarly, Van De Vijver et al. (2020) have also used spectral classifier SVM based on PCA to test and discriminate between affected and non-affected pixels of potato canopy infected by *Alternaria solani* and reported an accuracy of 92%. The investigation reported by Zhang et al. (2020) revealed that the SVM-based spectral reflectance ratio construction method was used for the assessment of rice blast severity at the heading stage with an accuracy of 83.87%. In this study, the classification of disease severity levels was also performed using PLS, SVM, RF, MARS and DNN models. The performance of MARS, PLS, RF and DNN were found optimum during calibration whereas they performed poorly during validation with less overall accuracy and kappa< 0.6. But when regression was performed all the models performed well for calibration and validation (R^2^ > 0.9). Also, the regression models seem to be balanced in terms of calibration and validation values as compared to classification results. As the number of classes were very high (total 10 classes), there were overlapping among the classes. This may be the reason behind the poor performance of classification as compared to regression.

In this study, we used vegetation indices and machine learning techniques for determining the severity of the rice leaf blast. Even though excellent results were obtained, additional study is required to confirm the findings on a larger spatial scale and to explore the model’s range of potential applications for other plant species and diseases too. The study is confined to non-imaging spectrometry approach only. This can be further extended to different low cost imaging sensors having suggested spectral bands from this study at air-borne and satellite platforms for upscaling to regional applications.

## Conclusion

The present study revealed that spectral data from the NIR region can be employed to assess stress-induced changes in the structure of plant tissue, such as cell collapse. The developed indices and multivariate models represented in the present study were able to predict different levels of disease severity caused by rice blast pathogens with very good accuracy and precision. The insight into the already existing indices expressed TVI to be the best one, whereas the proposed indices, RBI and NDBI performed better than other existing indices. The present findings also suggested the SVM as a robust approach for assessing the rice blast disease severity level. In the future, the methodology developed would help in its further use in high-throughput phonemics of different crops for biotic stresses and the development of a forewarning system for blast diseases. This would also help to frame suitable integrated disease management systems for different crops having infestation chances of blast diseases to attain a step forward towards agricultural sustainability.

## Data availability statement

The original contributions depicted in the present study are included in the article/supplementary material. Further inquiries can be directed to the corresponding author.

## Author contributions

NM, RS and DD conducted the investigation. NM, SA, RS, BD and SG implemented all the analysis. RS and DD conceptualized the study. NM, SA, RS, DD and AM prepared the original draft. HR, JM, AK and VC supervised the experiment. All authors contributed to the article and approved the submitted version.
